# Immunomodulatory Dual-Sized Microparticle System Conditions Human Antigen Presenting Cells Into a Tolerogenic Phenotype *In Vitro* and Inhibits Type 1 Diabetes-Specific Autoreactive T Cell Responses

**DOI:** 10.3389/fimmu.2020.574447

**Published:** 2020-10-22

**Authors:** Maigan A. Brusko, Joshua M. Stewart, Amanda L. Posgai, Clive H. Wasserfall, Mark A. Atkinson, Todd M. Brusko, Benjamin G. Keselowsky

**Affiliations:** ^1^Department of Pathology, Immunology, and Laboratory Medicine, University of Florida Diabetes Institute, Gainesville, FL, United States; ^2^J. Crayton Pruitt Family Department of Biomedical Engineering, University of Florida, Gainesville, FL, United States; ^3^Department of Pediatrics, University of Florida, Gainesville, FL, United States

**Keywords:** poly-lactic-co-glycolic acid, microparticle, autoimmunity, immunoregulation, combination therapy, type 1 diabetes

## Abstract

Current monotherapeutic agents fail to restore tolerance to self-antigens in autoimmune individuals without systemic immunosuppression. We hypothesized that a combinatorial drug formulation delivered by a poly-lactic-co-glycolic acid (PLGA) dual-sized microparticle (dMP) system would facilitate tunable drug delivery to elicit immune tolerance. Specifically, we utilized 30 µm MPs to provide local sustained release of granulocyte-macrophage colony-stimulating factor (GM-CSF) and transforming growth factor β1 (TGF-β1) along with 1 µm MPs to facilitate phagocytic uptake of encapsulated antigen and 1α,25(OH)_2_ Vitamin D_3_ (VD3) followed by tolerogenic antigen presentation. We previously demonstrated the dMP system ameliorated type 1 diabetes (T1D) and experimental autoimmune encephalomyelitis (EAE) in murine models. Here, we investigated the system’s capacity to impact human cell activity *in vitro* to advance clinical translation. dMP treatment directly reduced T cell proliferation and inflammatory cytokine production. dMP delivery to monocytes and monocyte-derived dendritic cells (DCs) increased their expression of surface and intracellular anti-inflammatory mediators. In co-culture, dMP-treated DCs (dMP-DCs) reduced allogeneic T cell receptor (TCR) signaling and proliferation, while increasing PD-1 expression, IL-10 production, and regulatory T cell (Treg) frequency. To model antigen-specific activation and downstream function, we co-cultured TCR-engineered autoreactive T cell “avatars,” with dMP-DCs or control DCs followed by β-cell line (ßlox5) target cells. For G6PC2-specific CD8^+^ avatars (clone 32), dMP-DC exposure reduced Granzyme B and dampened cytotoxicity. GAD65-reactive CD4^+^ avatars (clone 4.13) exhibited an anergic/exhausted phenotype with dMP-DC presence. Collectively, these data suggest this dMP formulation conditions human antigen presenting cells toward a tolerogenic phenotype, inducing regulatory and suppressive T cell responses.

## Introduction

Ideal immunotherapy approaches in antigen-specific autoimmune disease must abrogate autoimmunity without the need for broad and sustained systemic immunosuppression. In the case of type 1 diabetes (T1D), insulin is a primary target antigen during disease development and thus, also for tolerance induction to prevent disease onset (1, 2). Historically, attempts to tolerize against insulin have demonstrated exceptional safety profiles, yet failed to meet clinical endpoints in major T1D prevention trials (3–6). In the case of established T1D, therapeutic success will likely hinge on elimination of the autoreactive T cells presumably responsible for the destruction of pancreatic β-cells or external measures to control T cell behavior (7). Indeed, non-antigen-specific strategies targeting T cells have shown success in subjects with or at-risk for T1D, temporarily maintaining C-peptide production or delaying disease onset, but the decline in C-peptide and T1D progression eventually resumes, suggesting treated subjects do not develop lasting tolerance to islet antigens (8–14). The field has called for the use of combination therapies as a potentially more effective strategy to augment T cell targeted agents (15–19). To address this, we developed a novel biomaterial therapy to deliver immunomodulatory agents along with autoantigen as a means to recruit and tolerize dendritic cells (DCs) for robust antigen-specific T cell tolerance (20, 21). Here, we extensively characterized human immune cell responses *in vitro* as an important bridge to clinical translation for this novel dual sized microparticle (dMP) formulation.

DC-based therapies have gained interest in both cancer and autoimmunity due to the unique ability of DCs to direct inflammatory or anti-inflammatory T cell effector responses to their presented antigen (22–26). Many approaches center around the generation of antigen-specific presenting DCs *ex vivo*; however, high cost, impaired cell migration, and poor survival upon delivery complicate clinical translation (27, 28). Thus, we have pursued strategies to direct DC function *in vivo*. The intrinsic phagocytic capacity of DCs and other antigen presenting cells (APCs), as well as their abundant presence as immune sentinels in the epidermis (29), make them an attractive target for subcutaneously delivered payloads encapsulated in a particulate biomaterial (30, 31). Antigen-loaded particles are an area of particular interest in various disease models (28, 32, 33). Poly-lactic-co-glycolic acid (PLGA) has been well characterized as a delivery vehicle to DCs and is a component in multiple FDA-approved products (e.g., dissolvable sutures) (34). Previous work from our group and others demonstrated that PLGA microparticles (MPs) of approximately 1 μm in diameter are efficiently endocytosed for directed endosomal delivery, while particles 30 μm in diameter, too large to be taken up by APCs, provide controlled local release of encapsulated factors extracellularly to generate a tolerogenic milieu (35, 36). Our strategy involves combining a disease-relevant autoantigen with immunomodulatory agents selected for their ability to recruit DCs, create a suppressive APC phenotype, and induce durable antigen-specific T cell tolerance. We previously screened immunomodulatory agents of interest encapsulated in PLGA for their abilities to effect tolerogenic activity by murine bone-marrow derived DCs in mixed lymphocyte reactions. The resultant dMP was comprised of large MPs (30 μm) encapsulating transforming growth factor β1 (TGF-β1) and granulocyte-macrophage colony-stimulating factor (GM-CSF) for extracellular conditioning, along with small MPs (1 μm) containing 1α,25(OH)_2_ Vitamin D_3_ (VD3) and denatured insulin antigen for phagocytic engulfment (20, 36).

The tolerogenic capacity of the individual agents has been previously characterized in several settings. TGF-β1 is a potent immunoregulatory cytokine capable of suppressing effector function and cytokine production by both innate and adaptive immune cells (37). TGF-β1 treated DCs demonstrate reduced expression of MHC-II, co-stimulatory molecules, and inflammatory cytokines; increased production of the tolerogenic enzyme, indoleamine 2,3-dioxygenase (IDO) (38, 39); increased capacity for the induction of antigen-specific regulatory T cells (Tregs); and deletion of antigen-specific effector T cells (40). Additionally, as a critical mediator in differentiation and development of myeloid DCs, GM-CSF has been shown to promote DC recruitment in multiple disease applications (41), including the non-obese diabetic (NOD) mouse model of T1D where adoptive transfer of GM-CSF exposed DCs promoted the expansion of Foxp3^+^ Tregs and delayed diabetes onset (42). VD3 is well-known for its ability to steer DCs to a tolerogenic phenotype by inhibiting their maturation and promoting anti-inflammatory cytokine production, thus reducing T cell proliferation and effector response (43–47). Additionally, deficiencies in vitamin D, its receptor, and binding proteins have been found in multiple autoimmune and autoinflammatory conditions, including T1D, multiple sclerosis, rheumatoid arthritis, systemic lupus erythematosus, and Crohn’s disease (48–50). Thus, we designed our dMP formulation to impact multiple tolerogenic pathways active in innate and adaptive immune subsets for the induction of antigen-specific immune regulation.

This carefully selected combination of tolerogenic agents and disease-relevant autoantigen, delivered *via* PLGA MP encapsulation for subcutaneous injection, has been tested in two murine models of antigen-specific autoimmunity. This therapy successfully prevented diabetes in NOD mice and reduced disease severity in an early treatment model of experimental autoimmune encephalomyelitis (EAE) (21, 51). Often, efficacy in mouse models does not scale to trials in human subjects, highlighting the need for *in vitro* preclinical assays to test dose-response in target cells, as well as off-target or unexpected effects (52). Hence, we modeled biomaterial therapeutic responses to the immunomodulatory dMP agents in human subjects *via in vitro* culture and phenotyping of primary human monocytes, monocyte-derived DCs (hereafter referred to as DCs), primary T cells, and autoreactive T cell avatars engineered *via* T cell receptor (TCR) gene transfer (53) as a step toward supporting clinical translation.

## Materials and Methods

### MP Fabrication and Characterization

PLGA MPs were manufactured as previously described (51) with some noted modifications. Briefly, a 50:50 polymer composition of PLGA (molecular weight (MW) 44,000 g/mol; Corbion Purac), was used in a standard water-oil-water double solvent evaporation technique. Emulsions were formed with the aqueous phase comprised of Ultrapure H_2_O (Barnstead GenPure, Thermo Fisher Scientific). Poly-vinyl alcohol (PVA; MW approximately 15,000 g/mol; Fisher Scientific) was used as an emulsion stabilizer. To incorporate the desired protein(s), 100mg PLGA polymer was dissolved in methylene chloride (Fisher Scientific) at 5% w/v ratio. Protein solution containing either TGF-β1, GM-CSF, or VD3) was added to 5% PLGA solution and homogenized to form a primary emulsion. This emulsion was added to 2 mL of 5% PVA solution and homogenized to form the secondary emulsion. After transfer to a beaker containing 30 mL 1% PVA, resultant MPs were agitated using a magnetic stirrer for 4–6 h to evaporate residual methylene chloride. The remaining solution was centrifuged at 10,000x*g* for 10 min to collect MPs and washed 3x with Ultrapure H_2_O. MPs were then flash-frozen in liquid nitrogen, lyophilized, and stored at −20°C or −80°C until use. MP size distributions were measured using the Microtrac Nanotrac Dynamic Light Scattering Particle Analyser (Microtrac). Loading efficiency in MPs was measured using solvent extraction in DMSO followed by spectrophotometric analysis of protein content (51).

### Peripheral Blood Sample Collection and Processing

Following the provision of written informed consent, deidentified blood samples were collected from subjects without autoimmunity by venipuncture into sodium heparin coated Vacutainer tubes (BD) in accordance with University of Florida IRB201400709 and processed for leukocyte subsets *via* negative selection and Ficoll density gradient separation within 12 h of collection.

### Monocyte/Macrophage Culture

Peripheral blood mononuclear cells (PBMC) were isolated by density gradient centrifugation over Ficoll separation medium (GE) using established protocols. For monocyte/macrophage cultures, PBMC were incubated in 24-well tissue culture plates at 5x10^6^/mL in complete RPMI with 10% fetal bovine serum for 24 h, after which nonadherent cells were washed away to leave adherent monocytes. After MP incubation steps, cells were removed from tissue culture plastic *via* plate incubation on ice for 10 min and gentle scraping to avoid loss of surface marker expression potentially associated with protease treatment.

### Monocyte-Derived DC Generation and Culture

Monocytes were isolated from heparinized peripheral blood *via* negative selection (RosetteSep, StemCell) followed by density gradient centrifugation over Ficoll separation medium (GE). Monocytes were maintained at 10^6^/mL in DMEM (Gibco) with 10% heat-inactivated human serum and 50 ng/mL each of recombinant human GM-CSF and IL-4 (Peprotech) in ultra-low attachment plates (Corning) for 7-10 days. Small MPs (containing VD3 or equivalent mass unloaded PLGA, 5 µg) were incubated in wells with harvested DC in ultra-low attachment plates to allow for antigen/PLGA uptake. Large MPs (containing TGF-β1 and GM-CSF or equivalent mass unloaded PLGA) were added at 5 mg per 10^6^ DC in 0.4 μM pore size hanging well inserts (Miltenyi) to prevent cell overcrowding, for two days prior to testing for phenotype or stimulus response.

### Flow Cytometry

Antibodies for CD4 (clone RPA-T4, Biolegend), CD8 (clone RPA-T8, Biolegend), CD11c (clone Bly6, BD Biosciences), CD40 (clone 5C3, Biolegend), CD80 (clone 2D10, Biolegend), CD86 (clone BU63, Biolegend), HLA-DR (clone L243, Biolegend), Galectin 9 (clone 9M1-3, Biolegend), PDL1 (clone), CD25 (clone BC96, Biolegend), FOXP3 (clone 206D, Biolegend), ILT3 (clone ZM4.1, Biolegend), ILT4 (clone 42D1, Biolegend), PD1 (clone EH12, BD Biosciences), Eomes (clone WD1928, eBioscience), IDO (clone eyedio, eBioscience) were obtained, and cells were stained according to manufacturer-recommended protocols. Events were collected on an LSRFortessa cytometer (BD), and data were analyzed in FlowJo (Treestar). Representative gating strategies are presented in **Supplemental Figure 1**.

### Allogeneic T Cell Response Assay

CD4^+^ and CD8^+^ T cells were isolated from peripheral blood by negative selection (RosetteSep, StemCell) followed by density gradient separation. T cells were labeled with fluorescent proliferation dye [CellTrace Violet (CTV), Invitrogen] as per manufacturer’s instructions and cocultured with MP treated DC at a 1:1 ratio for up to 7 days to assess proliferation and effector function. In some experiments, memory (CD45RO^+^) and naïve (CD45RA^+^) T cells were FACS sorted (BD FACSAria) prior to DC coculture. Proliferation was quantified by gating the frequency of dividing cells, or by calculating the proliferation index (average number of divisions of responding cells; calculated in FlowJo as proliferation index = sum (i * N(i)/2^i^)/sum (N(i)/2^i^)where i = division number (undivided = 0) and N(i) = number of events in division i (54).

### Lentiviral Transduction and Generation of T Cell Avatars

Isolated CD4^+^ T cells were transduced with a multicistronic lentiviral TCR clone 4.13 as previously described (53). This clone reacts to GAD_555–567_ in the context of HLA-DRB1*04:01 and expresses an eGFP reporter on a pCNFW lentiviral vector backbone, driven by a cytomegalovirus (CMV) promoter. Isolated CD8^+^ T cells were transduced with a multicistronic lentiviral TCR expression construct encoding TCR clone 32, which recognizes the autoantigen glucose-6-phosphatase catalytic subunit 2 (G6PC2, formerly known as IGRP) in the context of HLA-A*02:01 (55, 56). In brief, T cells were resuspended in 1 mL of complete RPMI media (cRPMI) and cultured in a 24-well plate (250,000 cells/well). Cells were bead activated using anti-CD3 and anti-CD28 loaded Dynabeads (Life Technologies) at a 1:1 cell to bead ratio. After 48 h, protamine sulfate (Sigma) was added to a final concentration of 8 μg/mL. Lentivirus stock was added dropwise [multiplicity of infection (MOI) = 3]. Spinoculation was then performed by centrifuging the plate at 1000x*g* for 30 min at 32°C, followed by addition of IL-2 (200 U/mL) on days 2, 5, and 7.

### Supernatant Cytokine Analysis

Plates were centrifuged to remove cell debris, supernatants collected, and stored at -20°C until batch analysis of cytokines *via* Luminex multiplex bead assay (Millipore) or ELISA (BD OptEIA, BD Biosciences) as per manufacturer’s instructions.

### Antigen-Specific CD4^+^ T Cell Avatar Proliferation Assay

Seven days post-transduction, CD4^+^ T cell avatars (clone 4.13) were magnetically depleted of stimulation beads and flow-sorted (BD FACSAria) into GFP^+^ (*de novo* TCR^+^) and GFP^-^ (mock-transduced) populations prior to equilibration in IL-2 (50 U/mL) and IL-7 (10 ng/mL) for 5 days. Semi-quiescent cells were labeled with CTV proliferation dye for 1:1 co-culture with MP-treated HLA-DR4+ genotype selected (57) DCs (dye-labeled where indicated to ease gating strategies) in the presence of GAD_555–567_ peptide. Proliferation and expression of intracellular transcription factors, FOXP3 and Eomes, were assessed *via* flow cytometry at day 5.

### Antigen-Specific CD8^+^ T Cell Avatar Killing Assay

Previously cryopreserved CD8^+^ G6PC2-reactive TCR avatars (clone 32) were thawed and rested for 24 h with the homeostatic cytokine IL-7 (10 ng/ml) prior to coculture with MP-treated HLA-A2+ genotype selected (57, 58) DCs for 24 h in tissue culture treated plates at a 1:1 DC : CD8^+^ T cell ratio. Nonadherent CD8^+^ T cell avatars were removed from the plate, washed, and resuspended in fresh media for the killing assay. The human β-cell line, βlox5 (59, 60), was maintained under standard culture conditions, labeled with CTV fluorescent dye, and plated 18 h prior to the killing assay to achieve 80-90% confluency. At time of assay, media was removed and CD8^+^ T cell avatars were seeded at an effector to βlox5 target cell ratio of 5:1. Cell death was assessed at 18 h *via* flow cytometric analysis of Annexin V (BD Biosciences) and viability dye (Life Technologies) staining *via* an assay established in the lab (61).

### Statistical Analysis

Data were visualized using GraphPad Prism v.8 and analyzed by t tests or one-way ANOVA with multiple comparison testing as indicated in figure legends, with p < 0.05 considered significant.

## Results

### MP Characterization

**Figure 1:**
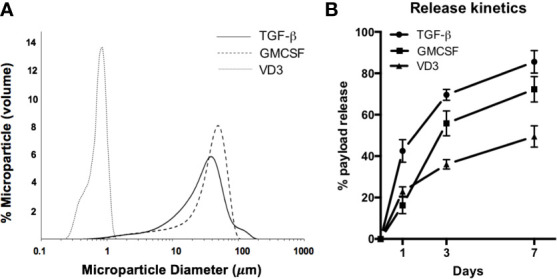
Characterization of MP formulation. **(A)** MP diameter was determined for small Vitamin D3-loaded MPs (dotted histogram), and large GM-CSF (dashed histogram) and TGF-β-loaded MPs (solid histogram). **(B)** Release of encapsulate into aqueous solution was assessed at 1, 3, and 7 days.

PLGA MPs were manufactured in a single large batch to provide standardization across *in vitro* experiments. MP size distribution, assessed by dynamic light scattering, showed that MPs exhibited the desired size characteristics (**Figure 1A**). Release kinetics in aqueous solution were determined at 1, 3, and 7 days to model payload release *in vitro* (**Figure 1B**). Encapsulation efficiencies were quantified *via* solvent extraction and spectrophotometric analysis, with TGF-β1 at 63.1 ± 6.6%, GM-CSF at 53.2 ± 7.3%, and VD3 at 73.4 ± 8.2% encapsulation efficiency. To control for biomaterial-induced cellular responses, *in vitro* assays were conducted with identical masses of drug-loaded PLGA MPs versus PLGA-only controls in each size class. These mass values, in combination with the release kinetics, were used to estimate *in vitro* concentrations of each agent at defined assay time points of 24 and 48 h (**Table 1**) and thereby, assess the effects of MP treatment on cellular phenotype and function.

**Table 1:** Encapsulation efficiency and effective microparticle dose.

Encapsulate	Input(ng/mg PLGA)	Encapsulation Efficiency (% ± SD)	MP mass *in vitro* (mg)	24 h dose (ng/mL)	48h dose (ng/mL)
rhTGF-β1	50	63.1 ± 6.6	5	67.1	88.5
rhGM-CSF	80	53.2 ± 7.3	5	34.6	76.8
Vitamin D3	100	73.4 ± 8.2	0.05	0.85	1.1

Encapsulation efficiencies were calculated via spectrophotometric analysis of microparticle (MP) batches. In vitro dosing was calculated based on the delivered MP mass and % release at defined time points.

### MP Responses in Isolated Cell Subsets

Previous work in the NOD mouse revealed a diverse immune cell composition surrounding the subcutaneous MP injection site, with neutrophils predominating in immediate response to PLGA material, followed by accumulation of the desired target of myeloid cells, as well as a significant number of T cells in response to drug-loaded dMP (20, 21). We therefore sought to investigate direct effects of dMPs on human cells belonging to those subsets specifically attracted by the dMP over PLGA—namely, monocytes/macrophages, DCs, and T cells—to assess therapeutic modulation.

#### Monocytes

**Figure 2:**
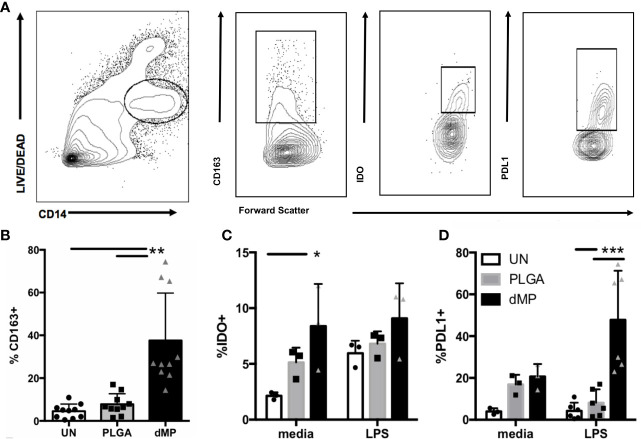
Monocyte MP response. **(A)** Gating schematic for live monocytes and indicated markers. **(B)** Expression of scavenger receptor CD163 on monocytes (n = 10) with media (white bars) PLGA MPs (gray bars) or full dMP (black bars). **(C)** Intracellular expression of IDO (n = 3) after incubation with PLGA or dMP (indicated as in **(B)**) shows increased frequency of IDO expression in media alone with dMP (left), to the level of the IDO-inducing stimulus LPS (right). **(D)** PD-L1 frequency (n = 6) increased significantly with dMP in the presence of LPS. Batch effects considered in analysis. (*p < 0.05, **p < 0.01, ***p < 0.001 by ANOVA with Tukey’s multiple comparison test).

The capacity of dMPs to induce phenotypic alterations in isolated human monocytes was investigated (**Figure 2**). Monocytes were incubated for 48 h under unstimulated conditions with media alone (UN) or both large and small empty MPs (PLGA) versus with MPs encapsulating dMP agents GM-CSF, VD3, and TGF-β1(dMP)) in the presence or absence of LPS for an additional 24 h of culture time (72 hr total). Tolerogenic markers of interest were assessed by flow cytometry of live CD14^+^ monocytes (representative gating in **Figure 2A**). dMP treated monocytes exhibited a significant increase (approximately 30%) in frequency of cells expressing the scavenger receptor CD163, a marker associated with anti-inflammatory M2 macrophages (62) (**Figure 2B**, dMP vs. UN and PLGA conditions, p < 0.01). Expression of the suppressive tryptophan-catabolizing enzyme, IDO, increased in response to dMP in the absence of LPS induction (**Figure 2C**, UN vs. dMP, p < 0.05), whereas PD-L1 was robustly induced in dMP treated cells above PLGA effects in in the presence of LPS stimulus (**Figure 2D**, dMP vs. UN/PLGA, p < 0.001). As expected, monocytes cultured with dMPs exhibited enhanced surface and intracellular expression of anti-inflammatory mediators.

#### DCs

**Figure 3:**
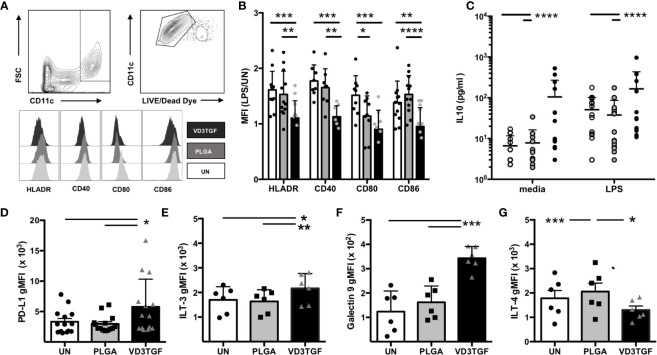
Dendritic cell modulation by dMP agents. (**A**, upper panels) Representative gating schematic for live CD11c^+^ DCs; (lower panels) representative histograms showing expression of activation markers HLA-DR, CD40, CD80, and CD86 under each culture condition (unstimulated (UN) in white, PLGA (gray) and VD3TGF (black) for gated DCs showing leftward shift in presence of dMP components. **(B)** Quantification of results depicted in **(A)** with calculated ratio of geometric mean fluorescence intensity (gMFI) in stimulated conditions (LPS) over unstimulated (UN) controls (n = 10) showing failure to upregulate activation markers in response to LPS with VD3TGF MPs in presence of GM-CSF. **(C)** IL-10 production in culture supernatants after (left) 72-h incubation with media (white circles), PLGA MPs (gray circles) or dMP (black circles), or 72-h incubation with treatments indicated as previous, with the addition of LPS (1 µg/ml) for the final 24 h of culture. dMP induced significantly increased IL-10 production over other treatments with or without inducing LPS stimulus. **(D–G)** Flow cytometry of replicate experiments for expression of negative regulators show increased intensity (gMFI) in presence of dMP for **(D)** PD-L1 (n = 14), **(E)** ILT-3 (n = 6), and **(F)** Galectin 9 with an apparent material associated increase in ILT-4 **(G)**. (*p < 0.05, **p < 0.01, ***p < 0.001, ****p < 0.0001 by ANOVA with Tukey’s multiple comparison test).

Human DCs were derived from peripheral blood monocytes *via* standard culture techniques and incubated for 48 h with large MP containing TGF-β1 and small MP containing VD3 (VD3TGF) versus PLGA and UN controls, in the presence of GM-CSF in culture media (**Figure 3**). GM-CSF containing MPs were not used in DC experiments as the effective MP dose (35 ng, **Table 1**) was lower than the media concentration necessary to induce and maintain DC differentiation (50 ng/mL). Following a 48 hour MP incubation, cells were treated with LPS (1 μg/mL) or media alone for an additional 24 h to assess response to inflammatory stimulus by flow cytometry, with representative gating schematic depicted for analysis of live DCs in **Figure 3A**. dMP treated DCs, hereafter referred to as dMP-DCs, showed a failure to upregulate canonical surface markers associated with antigen presentation (HLA-DR: dMP vs. UN, p < 0.001; dMP vs. PLGA, p < 0.01) and T cell costimulation (CD40: dMP vs. UN, p < 0.001; dMP vs. PLGA p < 0.01; CD80: dMP vs. UN, p < 0.001, dMP vs. PLGA, p < 0.05; CD86: dMP vs. UN, p < 0.01; dMP vs. PLGA, p < 0.0001) in the presence of LPS [calculated as mean fluorescence intensity (MFI) fold change] when compared to UN or PLGA controls (**Figure 3B**). This resistance to LPS-induced maturation and activation is characteristic of tolerogenic DCs. Supernatant analysis showed that dMP-DCs released significantly higher levels of IL-10 than both PLGA or UN controls, with LPS (**Figure 3C**, p < 0.0001) or media alone (p < 0.0001). Additionally, dMP-DCs exhibited increased expression levels of negative regulators as compared to UN or PLGA-DCs, namely PD-L1 (63) (**Figure 3D**, p < 0.05), ILT3 (64, 65) (**Figure 3E**, p < 0.05 and p < 0.01), and Galectin 9 (66) (**Figure 3F**, p < 0.001). ILT4 levels were slightly increased on PLGA-DCs (**Figure 3G**) compared to UN (p < 0.001) or dMP-DCs (p < 0.05). Altogether, these results suggest induction of a potent tolerogenic phenotype in dMP-DCs.

#### T Cells

**Figure 4:**
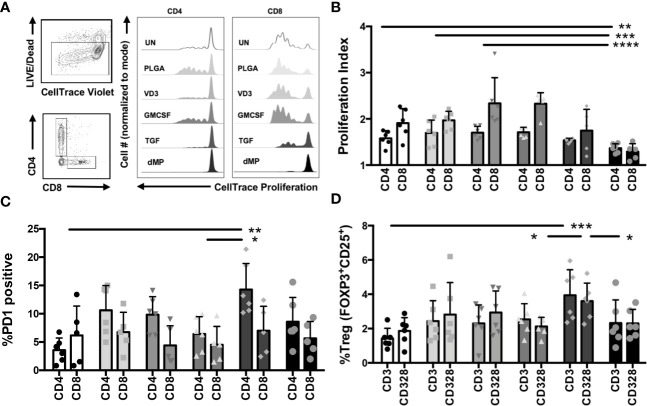
T cell proliferation and phenotype modulations with dMP. (**A**, left) Representative gating schematic for selecting live, CD4, or CD8 T cells for analysis after culture with single encapsulated dMP agents and complete dMP formulation; (right panels) representative histograms showing dye dilution indicating gated CD4 and CD8 T cell proliferation in each culture condition [unstimulated (UN, white), PLGA (light gray), individual agents VD3 (mid-gray), GM-CSF (dark gray), TGF (charcoal), and dMP (black)] showing reduced proliferation for TGF and dMP groups. **(B)** Quantification of results depicted in **(A)**, proliferation index for gated CD4 and CD8 T cells (n = 6 donors) with CD3/CD28 stimulation at 7 days post-stimulation with presence of indicated dMP agents. **(C)** %PD1 positive of gated CD4 T cells increased with TGFβ-MPs compared to UN and GM-CSF MPs. **(D)** % Treg of gated CD4 T cells increased with TGFβ-MPs compared to UN, GM-CSF, or dMP. (*p < 0.05, **p < 0.01, ***p < 0.001, ****p < 0.0001 by ANOVA with Tukey’s multiple comparison test).

Total CD3^+^ T cells were isolated from healthy control subjects by negative selection, proliferation dye-labeled, and treated for 48 h with single-component MP-encapsulated agents (GM-CSF, VD3, or TGF-β1) or the complete dMP formulation (GM-CSF, VD3, and TGF-β1) as compared to UN and PLGA controls. T cells were subsequently harvested from MPs *via* density-gradient centrifugation and cultured with plate-bound anti-CD3/anti-CD28 TCR stimulus for five days, whereupon cell proliferation and phenotype of both CD4^+^ and CD8^+^ T cells were assessed by flow cytometry (**Figure 4**). Representative gating and proliferation traces for CD4 and CD8 cells are shown (**Figure 4A**, representative of n = 6). Proliferation index (a measure of the division of responding cells) was significantly reduced in complete dMP-treated CD4 and CD8 T cells as compared to UN, PLGA, or individual agents (**Figure 4B**, dMP vs. UN, p < 0.01; dMP vs. PLGA, p < 0.001; dMP vs. GMCSF, p < 0.0001; dMP vs. VD3, p < 0.0001). Notably, proliferation of both CD4 and CD8 T cells was blunted by dMP with TCR stimulus (anti-CD3) and costimulatory signal (anti-CD28, shown) with similar results for cells treated with anti-CD3 only (not shown). Investigation of the mechanisms potentially underlying the observed reduced proliferation revealed that TGF-β1 MPs increased the frequency of PD1^+^CD4^+^ T cells compared to UN and VD3 MP treated T cells (p < 0.01), although PD1^+^CD8^+^ T cell frequencies were not significantly altered (**Figure 4C**). These findings demonstrate the interactions of individual MP components in the complete dMP in direct suppression of T cell activation and effector responses. Moreover, TGF-β1 MPs bolstered the frequency of Tregs among total CD4^+^ T cells (**Figure 4D**) as compared to UN (p < 0.0001), VD3 MP (p < 0.01), and dMP treated T cells (p < 0.01).

### Influence of MP Treatment on DC:T Cell Interactions

#### TCR Activation With dMP-DC

**Figure 5:**
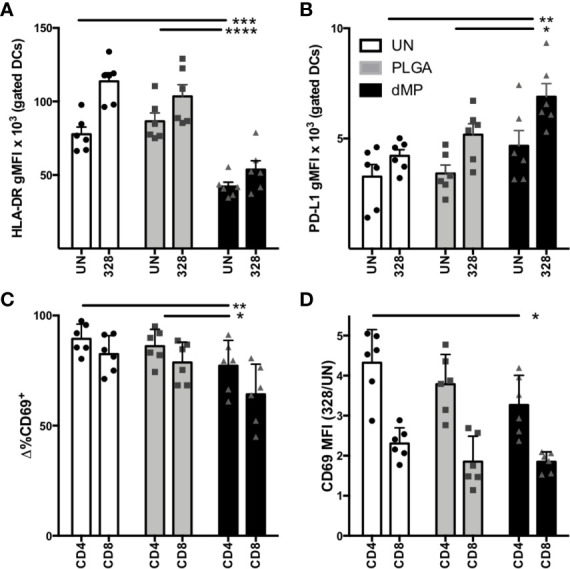
DC-T cell interactions after DC MP treatment. **(A)** DC expression of HLA-DR (n = 6) in T cell coculture after incubation with media alone [unstimulated (UN, white) PLGA (light gray), and dMP (black)] shows failure to upregulate HLA-DR and **(B)** upregulation of PD-L1 in response to T cell crosstalk. **(C)** Frequency of CD69^+^ cells in gated CD4 and CD8 populations decreased with dMP-DC coculture. **(D)** Upregulation of CD69 in response to TCR (CD3/CD28) stimulus (gMFI of stimulated divided by UN controls) for both CD4 and CD8 T cells decreased with dMP-DC incubation. (*p < 0.05, **p < 0.01, ***p < 0.001, ****p < 0.0001 by ANOVA with Tukey’s multiple comparison test).

With the observation of a tolerogenic phenotype in dMP-DCs, we next sought to confirm functional tolerogenic capacity *via* allogeneic human T cell co-culture. DCs (n = 3 donors) were proliferation dye labeled, then left untreated (UN-DCs), or conditioned with empty MPs (PLGA-DCs) or dMP (dMP-DCs) for 48 h, followed by MP removal *via* density gradient centrifugation. DCs from each donor were next co-cultured with proliferation dye-labeled T cells from two allogeneic donors (total experimental n = 6) at a 1:1 DC:T cell ratio. Cocultures (experimental n = 6) were incubated in media alone or with soluble CD3/CD28 TCR stimulus for T cell activation for 24 h, and examined by flow cytometry for both DC phenotype and T cell activation (**Figure 5**). dMP-DC response to T cell activation mirrored that seen with LPS stimulation (**Figure 3**). Namely, reduced HLA-DR expression was observed in response to dMP (**Figure 5A**, black bars) as compared to both UN-DCs and PLGA-DCs (p < 0.0001). PD-L1 expression was concomitantly increased in dMP-DCs as compared to UN-DCs (p < 0.01) and PLGA-DCs (**Figure 5B**, p < 0.05). Accordingly, both CD4^+^ and CD8^+^ T cells had significantly reduced frequency of cells expressing the TCR-activation marker, CD69, at 24 h post-stimulation in the presence of dMP-DCs (**Figure 5C**) compared to PLGA-DCs (p < 0.01) or UN-DCs (p < 0.01). CD4 T cell CD69 MFI was also significantly reduced with dMP-DC compared to UN-DC (**Figure 5D**, p < 0.01). These findings support the notion that dMP treatment blunts immediate TCR activation, giving the potential to influence downstream effector responses.

#### T Cell Phenotype and Function Post-Activation With dMP-DCs

**Figure 6:**
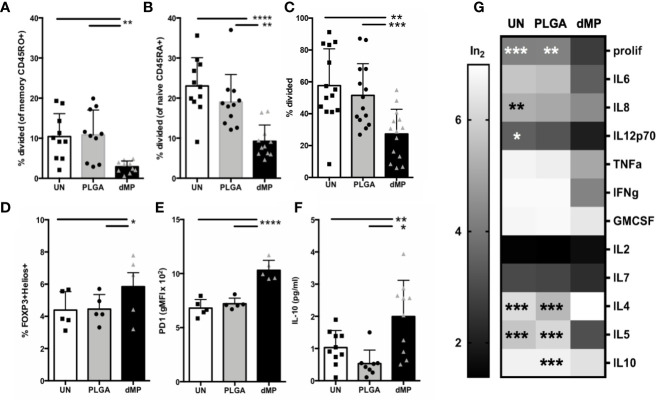
T cell activity and phenotype during coculture with MP-DCs. Proliferation of sorted **(A)** CD45RO^+^ memory T cells (n = 11) and **(B)** CD45RA^+^ naïve T cells (n = 10) was significantly suppressed in response to allogeneic dMP-DCs. **(C)** Proliferation of isolated total CD4 T cells (n = 14) was significantly suppressed in response to allogeneic dMP-DCs. **(D)** Increased presence of natural Tregs among total CD4 T cells (n = 5) was found in dMP-DC coculture. **(E)** PD-1 expression increased significantly with dMP-DCs (n = 5). **(F)** IL10 production in supernatant at day 5 was elevated (n = 9) with dMP-DCs. **(G)** A subset of T cell proliferation assays (displayed in **(C)**) were assessed for cytokines by multiplex bead assay and showed a general trend for decrease in cytokine production with T cell-dMP DC coculture (scaled for display and comparison by log transformation of raw values). % proliferation was included as a scale reference. Significance compared to dMP condition is indicated for individual outcome measures. (*p < 0.05, **p < 0.01, ***p < 0.001, ****p < 0.0001 by ANOVA with Tukey’s multiple comparison testing).

Downstream CD4^+^ effector T cell response to dMP-DCs was determined *via* co-culture of proliferation dye-labeled T cells with VD3-TGF-, PLGA-, or UN- pretreated allogeneic DCs at a DC:T cell ratio of 1:5 for 7 days. Proliferation and cellular phenotype were assessed by flow cytometry (**Figure 6**). dMP-DCs did not support alloantigen-induced proliferation of both CD45RO^+^ memory T cells (**Figure 6A**, dMP vs. UN and PLGA p < 0.01) and CD45RA^+^ naïve T cells at day 5 (**Figure 6B**, dMP vs. UN, p < 0.0001; dMP vs. PLGA, p < 0.01). In a subsequent experiment, a low dose of anti-CD3 (1 μg/mL) was added to induce TCR activation, leaving T cells reliant on DCs for costimulation. Again, dMP-DCs resulted in suppressed proliferation as compared to UN-DCs (p < 0.01) and PLGA-DCs (p < 0.001) (**Figure 6C**). Both FOXP3^+^Helios^+^ Treg frequency among CD4^+^ T cells (**Figure 6D**, dMP vs. UN/PLGA, p < 0.05) and PD1 expression on CD4^+^ T cells were increased in response to dMP-DCs at day 7 (**Figure 6E**, dMP vs. UN and PLGA, p < 0.0001). Despite reduced proliferation and therefore, lower total T cell number in all dMP-DC co-cultures, IL-10 production in culture supernatant was increased in cultures assessed at day 5 compared to PLGA- or UN-DC co-cultures (**Figure 6F**, dMP vs. UN, p < 0.05; dMP vs. PLGA, p < 0.01). Multiplex measurement of supernatant cytokines on day 7 (**Figure 6G**) revealed a trend toward decreased proinflammatory cytokines (IL-6, TNFα, IFNγ, GM-CSF, and IL7), a significant decrease in IL-8 (p < 0.01) and IL-12p70 (p < 0.05) as compared to UN-DC cocultures, and a significant decrease in IL-5 (p < 0.001) from both UN- and PLGA-DC cocultures. This decrease in T_H_1 and proinflammatory cytokines, with a concomitant significant increase in IL-4 compared to both control conditions (p < 0.001) and maintenance of IL-10 levels compared to untreated cultures, is suggestive of skewing to a T_H_2 phenotype. These alterations in T cell skewing were specific to the dMP, as PLGA-DC stimulus induced no significant changes in cytokine levels as compared to UN-DC. Given the reduced T cell proliferation, cytokine profiles were generally suppressed in presence of dMP, although production of IL-2 and IL-4 were enhanced in these cultures (p < 0.05). IL-10 production was elevated in response to dMP-DCs on day 5 when measured *via* ELISA (**Figure 6F**, p < 0.05), yet dMP-DC induced IL-10 was near UN-DC levels on day 7 with multiplex assay (**Figure 6G**), consistent with declining IL-10 levels over culture time for *in vitro* T cell stimulations (67).

#### Modeling of Antigen-Specific Effector T Cell Response to dMP

**Figure 7:**
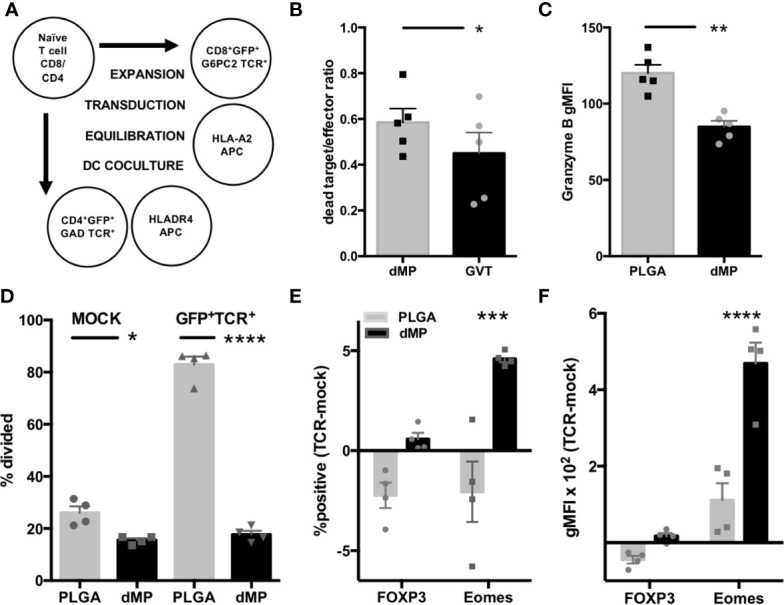
Assessment of antigen-specific T cell dMP response. **(A)** Schematic for generation of CD4 and CD8 T cell avatars *via* lentiviral transduction and coculture with dMP-treated HLA-selected APCs. **(B)** Results from target-killing assay for CD8 T cell avatars against the βlox5 beta cell line, with results represented as the ratio of dead labeled target cells to GFP^+^ T cells. **(C)** Expression of the cytotoxic effector protein Granzyme B in gated GFP^+^ T cells. **(D)** Proliferation in mock transduced T cells with allogeneic DC stimulus (left) was lower in presence of dMP, while antigen induced proliferation (right) was significantly controlled by dMP-APCs. **(E, F)** Analysis of intracellular staining of transcription factors associated with Treg (FOXP3) and T cell exhaustion (Eomes) showing difference of expression frequency with dMP pretreatment (**E**, % positive of gated GFP^+^ TCR^+^ cells − % positive in mock-transduced T cells) and difference of expression intensity (**F**, gMFI of gated GFP^+^ TCR^+^ cells – gMFI in mock-transduced T cells). (*p < 0.05, **p < 0.01, ***p < 0.001, ****p < 0.0001 by ANOVA with Tukey’s multiple comparison test).

CD4^+^ and CD8^+^ T cell avatars expressing TCRs reactive to the T1D autoantigens GAD (clone 4.13) and G6PC2 (clone 32), respectively, were generated from naïve T cells using lentiviral vectors as previously described (53). As TCR transduction requires T cell preactivation and expansion, sorted T cell avatars were equilibrated in homeostatic cytokines IL-2 and IL-7 for two days. This was followed by incubation for 24 h with nonadherent lymphocyte-depleted APCs from HLA-compatible donors, pretreated with PLGA or dMPs for 48 h (**Figure 7A**). CD8-32 avatars were assessed for phenotype and CTL function in killing assays using the lox5 human pancreatic β-cell line as target cells (55). dMP-APC conditioned CD8-32 cells were moderately suppressed in killinglox5 cells (**Figure 7B**, p < 0.05) and accordingly, exhibited significantly decreased expression of the serine protease Granzyme B in response to dMP-APCs versus PLGA-APCs, indicative of reduced cytotoxic potential (**Figure 7C**, 30% decrease in gMFI, p < 0.01). While untransduced GFP^-^CD4^+^ T cells (mock transduced) showed somewhat reduced proliferation in response to dMP-APC with allogeneic stimulus, CD4-GAD4.13 avatars showed dramatically reduced proliferation in response to dMP-APCs with exogenous GAD_555–567_ peptide stimulus (GFP^+^, 75% decrease in proliferating cells; p < 0.0001, **Figure 7D**). Modest increases in the frequency of FOXP3^+^ cells and FOXP3 gMFI were observed in dMP-APC activated CD4^+^ T cell avatars following exposure to autoantigen, whereas dMP-APC activation significantly increased frequency (p < 0.01) and expression intensity (4.5-fold increase, p < 0.0001) of the exhaustion-associated transcription factor Eomes (**Figures 7E, F**) (68, 69). Altogether, these data indicate dMP-APCs are capable of strongly directing T1D antigen-specific CD4^+^ T cell responses, with more modest control over CD8^+^ T cells.

## Discussion

In development and testing this dMP platform, we implemented a sequential iterative strategy of *in vitro* screening with formulation testing in mouse immune cells and validation of *in vivo* efficacy in two murine models of autoimmunity (21, 51), followed by both replicative *in vitro* experiments and discovery efforts in human cells as reported herein. We validated target immune effects using standard immune function assays, then modeled autoimmune responses to evaluate indirect immunomodulation of engineered autoreactive T cells exposed to dMP-APCs. These data support the clinical translatability of this dMP formulation, as therapeutic goals defined and met in animal models of autoimmunity (21, 51) were recapitulated *in vitro* in human cells.

Notably, dMP treatment of both monocytes and monocyte-derived DCs robustly induced a suppressive cell phenotype and dampened responses to inflammatory stimulus. Human dMP-DC results were analogous to those seen in murine bone marrow derived DCs (20, 36), with marked resistance to LPS-induced maturation after brief incubations with soluble GM-CSF plus MP encapsulated TGF-β1 and VD3. dMP-DCs displayed a comprehensive suppressive phenotype, with high expression of multiple immunoregulatory markers (PD-L1, ILT3, Galectin 9) and demonstrated ability to restrain T cell responses. Expression of ILT3, induced on DCs *in vitro* by IL-10 in combination with IFN or VD3, influences unprimed T cells to become suppressive (70). Induction of ILT3 and PD-L1 on DCs has been shown with individual agents in the dMP formulation, namely VD3 (45, 47, 71, 72). PD-L1^+^ DCs are known to restrain T cell activation, differentiation, and proliferation during priming (73, 74). Administration of Galectin 9 at supraphysiological levels has shown promise in suppressing T_H_1 inflammation, (66), promoting Treg (75), and preventing T1D development in the NOD mouse *via* islet expression (76). Additionally, Galectin 9 expression on DCs assists their migration toward chemokines (77), thus suggesting that dMP treatment could enhance DC migratory capacity from the injection site to draining lymph nodes. Importantly, dMP-DCs maintained their desirable tolerogenic surface marker profile following both LPS stimulus and T cell crosstalk post-anti-CD3/CD28 activation. T cells responding to dMP-DCs exhibited reduced immediate TCR activation and downstream proinflammatory responses. Notably, these data indicate that dMP exposure influenced T cell skewing, with a major shift toward a T_H_2 phenotype characterized by high levels of IL-4 production. The skewing of cytokine production after exposure to dMP-DCs versus PLGA-DCs or UN-DCs is consistent with lower effective TCR signal strength (78, 79), biasing T cell differentiation toward a T_FH_/T_H2_ phenotype.

This work included assessment of dMP influence on human monocytes/macrophages, which were abundantly present at the MP injection site *in vivo* in the NOD mouse (21) and could provide additional local immunoregulation beyond the tolerogenic DC and cytokine milieu promoted by dMP. The potential for dMP-monocytes to induce robust T cell suppression was demonstrated, whereas HLA-matched donor monocytes were not differentiated to DCs prior to incubation with dMP and subsequent T cell co-culture. In addition to DCs, monocytes and macrophages could provide additional antigen-specific immunoregulation of T cells encountered (80) and deletion of autoreactive T cells in peripheral and inflamed sites through the observed increased IDO expression (81). Indeed, M2 macrophages have been shown to promote tolerance of transplant tissue and suppression of xenoimmune response *via* IDO expression and production of TGF-β1 (82, 83). Additionally, human monocytes have a unique ability to activate latent TGF-β1 on surrounding cells, providing a potential feedforward loop for this potent suppressive mediator (84).

We measured T cell responses following direct culture with the dMP formulation. In agreement with the prior literature, TGF-β1 MPs alone drove induction of FOXP3 and PD-1 (85), whereas only the full dMP provided both significant reduction in TCR-induced cellular proliferation and inflammatory cytokine production. Our data indicate that in addition to indirect suppression by interactions with dMP-tolerized DCs in lymph nodes, T cell phenotype and activity could be directly influenced at the injection site through mechanisms of infectious tolerance demonstrated in TGF-β1 producing T_H_3 peripheral Tregs (86). Notably, while the specific dMP formulation is tunable for disease-specific applications, the combination of agents in the dMP tested here synergized to influence T cell effector function. This may provide additional benefit in the case of antigen-specific T cell localization to a peripheral antigen depot, as recently reported in an antigen-defined T1D mouse model (87).

Finally, we assessed the impact of the dMP on antigen-specific T cell responses to two β-cell autoantigens in T1D. We utilized our established platform for generating T cell avatars by multi-cistronic TCR expression to assess dMP-mediated suppression of islet antigen-specific T cell activity (53). With this platform, we previously demonstrated that autoreactive T cells may play a direct role in β-cell killing, as G6P2C-specific CD8^+^ cytotoxic T lymphocyte avatars are able to directly lyse both β-like cell lines and primary human β-cells (55). Moreover, our studies of human tissues from the Network for Pancreatic Organ donors with Diabetes (nPOD) have demonstrated that clone 4.13, originally identified in T1D PBMC (88), can be found within the islets of a human T1D pancreas (89). Hence, this system represents an excellent platform for preclinical translation. dMP-DCs were able to potently suppress the proliferation and drive anergy in CD4^+^ GAD4.13 T cell avatars, and though dMP-DCs reduced Granzyme B expression by G6PC2 avatars, they only moderately reduced cytotoxic effects. These results suggest that our dMP formulation, despite decreasing effector molecule expression and inducing markers of T cell exhaustion, is not capable of fully reversing the cytotoxic activity of an activated CTL with high affinity TCR. For optimal reversal of T1D in human subjects, these results suggest potential opportunity for pretreatment with a depleting agent, such as anti-CD3 (teplizumab) or anti-thymocyte globulin (ATG, thymoglobulin) prior to reconditioning the host with this tolerogenic dMP vaccine (90).

The experiments conducted herein further support the development of antigen-specific tolerogenic vaccine strategies that leverage the many benefits of controlled release from biocompatible MPs. Experimental conditions described herein were meant to model the effect on cells encountering the persistent injection site microparticle depot induced by dMP injection, wherein the local concentration of agents released by microparticles is high, followed by trafficking to draining LN to present ingested antigen.

Here, we expand the scope of the dMP from preventing and reversing T1D in the NOD mouse (20, 21) and ameliorating EAE in a mouse model of multiple sclerosis (51) to illustrate efficacy in human cells, notably using autoreactive T cell clones specific for two T1D-associated antigens. Importantly, the dMP system is modular in nature and could be adapted to include additional antigens and epitopes that continue to emerge as targets of autoreactive T cells in T1D (91, 92). In summary, the use of bioengineering approaches, including the dMP formulation reported here, offers the capacity to safely deliver effective vaccines to intervene in the disease process of chronic autoimmune conditions.

## Data Availability Statement

The raw data supporting the conclusions of this article will be made available by the authors, without undue reservation.

## Ethics Statement

The studies involving human participants were reviewed and approved by University of Florida Institutional Review Board. Written informed consent to participate in this study was provided by the participant or in the case of minors, the participants’ legal guardian/next of kin.

## Author Contributions

MAB conceived the study, designed and performed the experiments, analyzed the data, and wrote the manuscript. JMS designed the experiments, manufactured the microparticles, and wrote the manuscript. ALP wrote the manuscript. CHW, MAA, and TMB contributed intellectually and wrote the manuscript. BGK conceived and directed the study, and wrote the manuscript. All authors contributed to the article and approved the submitted version. As guarantor of this work, BGK assumes full responsibility for the ethical collection, analysis, and reporting of the data and the decision to publish.

## Funding

This work was supported by the National Institutes of Health (R01 DK091658, R01 DK098589, R01 DE027301, and R01 AI133623 to BGK; T32 DK108736 to JMS; and P01 AI042288 to MAA and TMB), the University of Florida Clinical and Translational Science Institute Network Science Pilot award, and the McJunkin Family Charitable Foundation, Inc.

## Conflict of Interest

BGK, CHW, TMB, and MAA are cofounders of OneVax, LLC, a preclinical biotechnology company with interest in the dMP technology.

The remaining authors declare that the research was conducted in the absence of any commercial or financial relationships that could be construed as a potential conflict of interest.

## Supplementary Material

Supplementary Figure 1 Gating of T cell subset and functional markers. Representative flow data and gating strategy for the identification of **(A)** PD-1 on dividing CD4 and CD8 T cells in **Figure 4**, **(B)** FOXP3^+^CD25^+^ regulatory T cells as a distinct subset from activated CD4^+^CD25^+^ T cells in **Figure 4**, **(C)** CD69^+^ cells in **Figure 5**, and **(D)** FOXP3^+^Helios^+^ natural Treg in **Figure 6**.

## Abbreviations

PLGA, poly-lactic-co-glycolic acid; dMP, dual-sized microparticle; GM-CSF, granulocyte-macrophage colony-stimulating factor; TGF-β1, transforming growth factor β1; VD3, 1α,25(OH)_2_ Vitamin D_3_; T1D, type 1 diabetes; EAE, experimental autoimmune encephalomyelitis; DCs, dendritic cells; dMP-DCs, dMP-treated DCs; TCR, T cell receptor; Treg, regulatory T cell; APCs, antigen presenting cells; IDO, indoleamine 2,3-dioxygenase; NOD, non-obese diabetic; MW, molecular weight; PVA, Poly-vinyl alcohol; PBMC, Peripheral blood mononuclear cells; CTV, CellTrace Violet; CMV, cytomegalovirus; cRPMI, complete RPMI media; MOI, multiplicity of infection; UN, media alone unstimulated conditions; VD3TGF, large MP containing TGF-β1 and small MP containing VD3; MFI, mean fluorescence intensity.
