# Supplementary figures and images for: Immunomodulatory Dual-Sized Microparticle System Conditions Human Antigen Presenting Cells Into a Tolerogenic Phenotype In Vitro and Inhibits Type 1 Diabetes-Specific Autoreactive T Cell Responses
Source: Front Immunol. 2020 Oct 22;11:574447. doi: 10.3389/fimmu.2020.574447 (PMC7649824; doi:10.3389/fimmu.2020.574447)

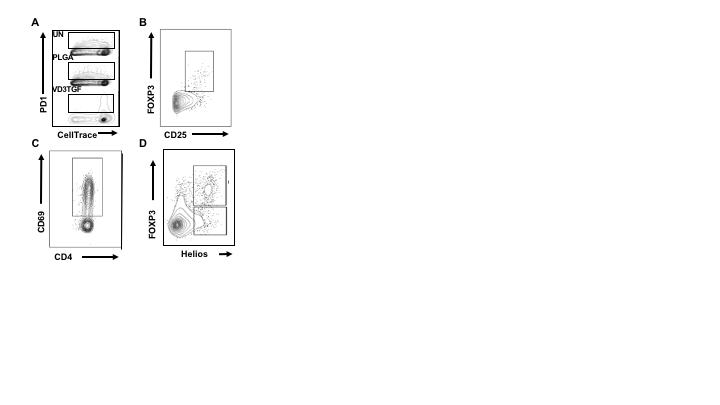

Supplement: Supplementary Figure 1 — Gating of T cell subset and functional markers. Representative flow data and gating strategy for the identification of (A) PD-1 on dividing CD4 and CD8 T cells in Figure 4, (B) FOXP3+CD25+ regulatory T cells as a distinct subset from activated CD4+CD25+ T cells in Figure 4, (C) CD69+ cells in Figure 5, and (D) FOXP3+Helios+ natural Treg in Figure 6. [file Image_1.tiff]
